# Association between a dietary index for gut microbiota and breast cancer history in adult women: findings from NHANES 2011–2020

**DOI:** 10.3389/fnut.2025.1619809

**Published:** 2025-06-17

**Authors:** Zhiwei Fan, Liang Li, Lingling Bu, Zhihui Geng, Song Liu

**Affiliations:** ^1^Department of Thyroid and Breast Surgery, The Affliated Taihe Hospital of Traditional Chinese Medieine, Anhui University of Chinese Medieine, Taihe, China; ^2^Department of Head and Neck, Breast and Thyroid Surgery, The First Affiliated Hospital, University of Science and Technology of China, Hefei, China

**Keywords:** breast neoplasms, diet, gut microbiota, nutrition surveys, NHANES

## Abstract

**Background:**

Gut microbiota plays a crucial role in cancer development, yet limited studies have explored microbiota-oriented diets in relation to breast cancer risk. The aim was to investigate the association between a gut microbiota–oriented dietary index (DI-GM) and breast cancer risk among U.S. women.

**Methods:**

This cross-sectional study analyzed data from 6,083 women aged ≥20 years from NHANES 2011–2020. The DI-GM score, based on intake of microbiota-beneficial and microbiota-unfavorable foods, was constructed from 24-h dietary recalls. Breast cancer history was self-reported. Multivariable logistic regression models were used to examine associations. Subgroup analyses assessed potential effect modification.

**Results:**

Higher DI-GM scores were significantly associated with lower odds of breast cancer (adjusted OR = 0.94, 95% CI: 0.89–0.99, *p* = 0.012). Women with DI-GM scores ≥6 had a 33% lower likelihood of breast cancer compared to those with lower scores (adjusted OR = 0.67, 95% CI: 0.45–0.89, *p* = 0.006). Subgroup analyses showed consistent associations across age, ethnicity, smoking, alcohol, and BMI categories without significant interactions.

**Conclusion:**

Following a diet that supports a healthy gut microbiota may help reduce the risk of breast cancer. Additional longitudinal and mechanistic research is needed to validate these results.

## Introduction

Breast cancer is the most commonly diagnosed cancer among women in the United States, excluding skin cancers. In 2025, it is estimated that approximately 316,950 new cases of invasive breast cancer will be diagnosed in women, along with 59,080 cases of ductal carcinoma *in situ* (DCIS). Furthermore, about 42,170 women are expected to die from breast cancer this year. The average lifetime risk for a woman in the U.S. developing breast cancer is approximately 13%, or 1 in 8 (1). While established risk factors such as age, genetic predisposition, hormonal influences, and lifestyle behaviors contribute to breast cancer development (2, 3), emerging evidence suggests that the gut microbiota may also play a significant role in modulating breast cancer risk (4).

A systematic review and meta-analysis revealed that women with breast cancer exhibited reduced gut microbial diversity compared to healthy controls, suggesting a potential link between microbial composition and carcinogenesis (5). The gut microbiota influences breast cancer risk through multiple mechanisms. It modulates estrogen metabolism via bacterial enzymes like β-glucuronidase, increasing circulating estrogen levels that can promote hormone receptor–positive breast cancer (6). Additionally, gut dysbiosis can trigger systemic inflammation and immune dysregulation, creating a pro-tumorigenic environment that facilitates breast cancer development and progression (7).

Numerous studies have examined individual dietary components and their effects on gut microbiota and subsequent cancer risk (8–10). The recently developed Dietary Index for Gut Microbiota (DI-GM) addresses this gap by integrating 14 dietary components, such as fermented dairy, chickpeas, whole grains, and fiber that have been shown to beneficially influence gut microbiota composition, as well as components like red and processed meats that may have adverse effects (11). Various studies have investigated the association between DI-GM and health conditions such as metabolic dysfunction (12), diabetes (13), liver fibrosis (14), etc. As our knowledge, there is no study investigating the association between DI-GM and breast cancer risk. Therefore, applying the DI-GM to large, nationally representative datasets like the National Health and Nutrition Examination Survey (NHANES) could provide novel insights into the relationship between diet, gut microbiota, and breast cancer risk, potentially informing targeted dietary recommendations for prevention. The current study aims to elucidate potential association between DI-GM and breast cancer risk utilizing data from NHANES 2011–2020.

## Methods

### Study design and population

This cross-sectional study utilized publicly available data from the NHANES cycles 2011–2020. NHANES is a nationally representative program conducted by the National Center for Health Statistics (NCHS) that employs a complex, multistage, stratified probability sampling design to assess the health and nutritional status of the U. S. population.^1^ Female participants aged 20 years and older with complete information on dietary intake, breast cancer history, and relevant covariates were included in the analysis. Women who were pregnant at the time of the survey or had missing data on primary variables of interest were excluded to minimize potential bias. NHANES data are publicly available and de-identified; therefore, this study was exempt from institutional review board (IRB) approval. All participants provided written informed consent (15).

### Assessment of dietary intake and construction of DI-GM

**Figure 1:**
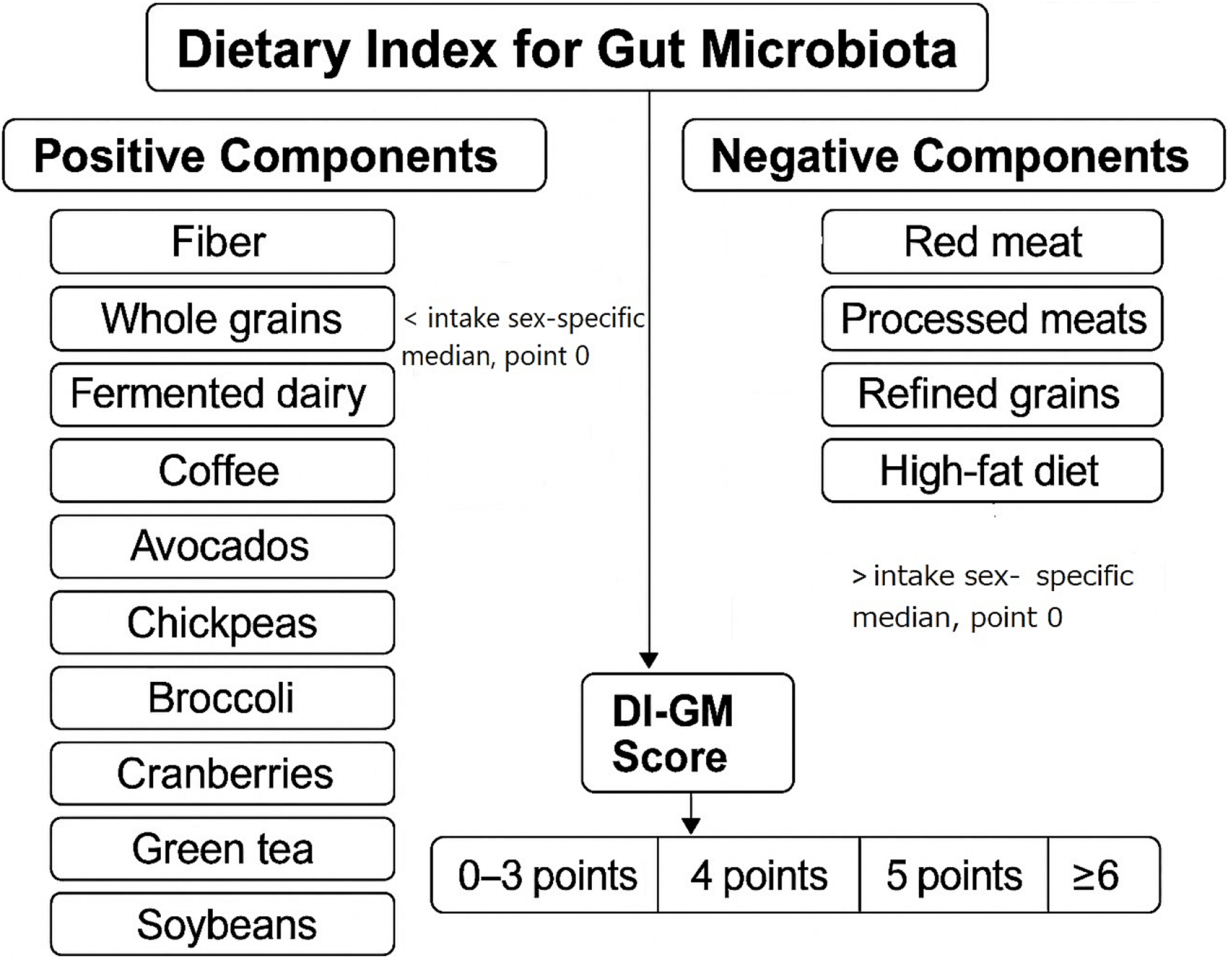
Scoring methodology for the dietary index for gut microbiota (DI-GM).

Dietary intake data were collected using two 24-h dietary recalls administered by trained interviewers following the United States Department of Agriculture (USDA) Automated Multiple-Pass Method. The validity and reliability of the 24-h recall method have been validated in previous research (16, 17). To assess diet quality in relation to gut microbiota health, we calculated the average intake of each dietary component across the two recalls and applied the DI-GM, a validated scoring system based on 14 key dietary components identified in the literature for their effects on gut microbiota composition. Components positively associated with gut microbiota health, including fiber, whole grains, fermented dairy, coffee, avocados, chickpeas, broccoli, cranberries, green tea, and soybean were assigned positive scores, while components negatively associated, such as red and processed meats, refined grains, and high-fat diet (40% of total caloric intake) were inversely scored. For each positive-associated food item, intake higher than gender-specific median led to a 1 positive point, otherwise 0. For each negative-associated food item, intake higher than gender-specific median led to a 0 point, otherwise 1. Each participant received a cumulative DI-GM score, with higher scores reflecting a diet more favorable to gut microbiota diversity and function (Figure 1) (11). Participants finally were categorized into four groups: first as 0–3, second as 4, third as 5, and fourth as ≥6 points.

### Assessment of breast cancer

Breast cancer status was determined based on participants’ self-reported response to the question, “Has a doctor or other health professional ever told you that you had breast cancer?” Participants who answered “yes” were classified as having a history of breast cancer.

### Covariates

Several covariates were included based on known associations with breast cancer risk and dietary patterns. These included age, race/ethnicity (Hispanic, Non-Hispanic White, Non-Hispanic Black, and Other), poverty-income ratio (PIR) [low (≤1.3), medium (>1.3 to 3.5), and high (>3.5) according to US government standards (18)], marital status (married/living with partner other), smoking status (current smoker, non-smoker), alcohol drinking alcohol drinking (0 g/d, 0.1 to 13.9 g/d, and ≥ 14 g/d), hypertension status (yes or no), and body mass index (BMI, kg/m^2^). Data on these variables were obtained through standardized NHANES interviews and physical examinations. Marital status was categorized as married/living with a partner or other.

### Statistical analysis

All analyses accounted for the NHANES complex sampling design by applying appropriate sampling weights, strata, and primary sampling units to produce nationally representative estimates. Characteristics of participants with and without a history of breast cancer were compared using independent-sample t-tests for continuous variables and chi-square tests for categorical variables. Multivariable logistic regression models were used to estimate odds ratios (ORs) and 95% confidence intervals (CIs) for the association between DI-GM score and breast cancer risk. In the primary analysis, DI-GM was modeled both as a continuous variable and in categories (0–3, 4, 5, and ≥6). Model 1 was unadjusted, while Model 2 adjusted for age, ethnicity, PIR, hypertension, BMI, and smoking status. The trend across DI-GM categories was assessed by modeling the median value within each category as a continuous variable. Associations between the beneficial and unbeneficial components of the DI-GM and breast cancer risk were also examined separately. Subgroup analyses were conducted to investigate potential effect modification by age (<40, 40–59, ≥60 years), ethnicity, smoking status, and BMI categories (<24.9, 25–29.9, ≥30 kg/m^2^). Interaction terms were included in regression models, and *p*-values for interaction were reported. To evaluate multicollinearity, we calculated the Variance Inflation Factor (VIF) for each covariate included in our regression models. All VIF values were below the commonly accepted threshold of 5, indicating that multicollinearity was not a significant issue in our models. All analyses were performed using Stata version 17.0 (StataCorp LLC, College Station, TX). A two-tailed *p*-value <0.05 was considered statistically significant.

## Results

### Characteristics of the study participants

Table 1 presents the characteristics of the study population. A total of 6,083 participants were included in the analysis, of whom 226 (3.7%) reported a history of breast cancer. The mean age of the population was 53.6 years. The overall mean score for the DI-GM was 5.4. Compared to individuals without breast cancer, those with a history of breast cancer were more likely to be older, of Non-Hispanic White ethnicity, and to have a higher prevalence of hypertension (all *p* < 0.001). Participants with breast cancer also had significantly higher BMI and lower DI-GM scores, particularly in the beneficial component of the index (all *p* < 0.01).

**Table 1:** Characteristics of the study participants, NHANES (2011–2020).

Characteristic	Overall (*N* = 6,083)	Non-breast cancer (*N* = 5,857)	Breast cancer (*N* = 226)	*P*-value
Age (years)	53.6 ± 11.7	53.2 ± 11.6	60.8 ± 10.5	**<0.001**
PIR (%)				
<1.30	1,402 (23.0)	1,350 (23.0)	52 (23.0)	**0.065**
1.30–3.50	3,089 (50.8)	2,970 (50.7)	119 (52.7)	
≥3.50	1,592 (26.2)	1,537 (26.3)	55 (24.3)	
Ethnicity (%)				
Hispanic	1,143 (18.8)	1,124 (19.2)	19 (8.4)	**<0.001**
Non-Hispanic White	2,954 (48.6)	2,790 (47.6)	164 (72.6)	
Non-Hispanic Black	1,305 (21.5)	1,288 (22.0)	17 (7.5)	
Other	681 (11.2)	655 (11.2)	26 (11.5)	
Marital (%)				
Married/living with partner	3,996 (65.7)	3,849 (65.7)	147 (65.0)	0.461
Other	2,087 (34.3)	2,008 (34.3)	79 (35.0)	
Smoking status (%)				
Smoker	1,720 (28.3)	1,661 (28.4)	59 (26.1)	0.52
Non-smoker	4,363 (71.7)	4,196 (71.6)	167 (73.9)	
Alcohol drinking (%)				
Non-drinking	4,866 (80)	4,682 (79.9)	184 (81.4)	0.413
Low to moderate drinking	365 (6)	355 (6.1)	10 (4.4)	
Heavy drinking	852 (14)	820 (14)	32 (14.2)	
Hypertension (%)				
Yes	2,466 (40.5)	2,315 (39.5)	151 (66.8)	**<0.001**
No	3,617 (59.5)	3,542 (60.5)	75 (33.2)	
Body mass index (kg/m2)	29.1 ± 6.7	28.8 ± 6.6	31.4 ± 7.0	**<0.001**
DI-GM, mean ± SD	5.4 ± 1.5	5.5 ± 1.5	4.9 ± 1.4	**<0.001**
Beneficial to gut microbiota, mean ± SD	2.7 ± 0.9	2.8 ± 0.9	2.1 ± 0.8	**0.003**
Unbeneficial to gut microbiota, mean ± SD	2.7 ± 1.0	2.7 ± 1.0	2.8 ± 1.1	0.120

All continuous variables are presented as means (standard deviations) and categorical variables presented as numbers (%). *P*-values were calculated based on independent sample *t*-test or Chi-square test. DI-GM, dietary index for gut microbiota; NHANES, National Health and Nutrition Examination Survey; PIR, Poverty Income Ratio. PIR ≤1.30 indicates low income, which often qualifies individuals for federal assistance programs. PIR >1.30 to ≤3.50 represents middle income (131–350% of the poverty level), and PIR >3.50 denotes high income (>350% of the poverty level). Bold values indicate *p* < 0.05.

### Association between DI-GM and breast cancer

As shown in Table 2, higher DI-GM scores were inversely associated with history of breast cancer. In the unadjusted model, the DI-GM was significantly associated with reduced breast cancer odds (OR = 0.92, 95% CI: 0.87–0.97, *p* = 0.008), and this relationship remained significant after adjusting for covariates (adjusted OR = 0.94, 95% CI: 0.89–0.99, *p* = 0.012). Stratification by DI-GM categories revealed that participants with a score of ≥6 had a significantly lower likelihood of breast cancer (crude OR = 0.65, 95% CI: 0.54–0.85, *p* = 0.002; adjusted OR = 0.67, 95% CI: 0.45–0.89, *p* = 0.006), with a significant trend across increasing DI-GM levels (adjusted *p* for trend = 0.014). For the individual components of DI-GM, the beneficial subscore was inversely associated with breast cancer in both crude (OR = 0.87, 95% CI: 0.75–0.97, *p* = 0.016) and adjusted models (OR = 0.89, 95% CI: 0.78–0.99, *p* = 0.047).

**Table 2:** Association between DI-GM and breast cancer, NHANES 2011–2020.

Exposures	Model 1∗		Model 2 ∗∗	
	OR [95%CI]	*P*-value	OR [95%CI]	*P*-value
DI-GM	0.92 (0.87–0.97)	0.008	0.93 (0.88–0.99)	0.015
0–3	1.00 (reference)	–	1.00 (reference)	–
4	0.83 (0.66–1.05)	0.126	0.86 (0.61–1.17)	0.324
5	0.78 (0.58–0.97)	0.018	0.78 (0.6–1.06)	0.083
≥6	0.65 (0.54–0.85)	0.002	0.67 (0.45–0.88)	0.007
P for trend		0.004		0.017
Beneficial to gut microbiota	0.87 [0.75–0.97]	0.016	0.89 [0.77–0.99]	0.044
Unbeneficial to gut microbiota	1.10 [0.99–1.22]	0.079	1.08 [0.95–1.21]	0.216

*Model 1 not adjusted. **Model 2 adjusted for age, ethnicity, PIR, hypertension, body mass index, alcohol drinking, and smoking. DI-GM, dietary index for gut microbiota; NHANES, National Health and Nutrition Examination Survey; OR, Odds Ratio; CI, Confidence Interval.

### Subgroup analyses

Subgroup analyses were conducted to assess whether the association between DI-GM (as both a continuous and categorical variable) and breast cancer was modified by selected factors, including age, ethnicity, alcohol drinking, smoking status, and BMI (Table 3). Across all subgroups, an inverse relationship between DI-GM and breast cancer was observed. The association was most pronounced among participants aged ≥60 years (OR = 0.57, 95% CI: 0.42–0.79, *p* = 0.004), with similar trends evident among Non-Hispanic White people, non-drinkers, and non-smokers. However, after adjusting for relevant confounders, no statistically significant interactions were found across any of the examined subgroups (all *P* for interaction > 0.05).

**Table 3:** Associations between DI-GM and breast cancer, stratified by selected factors, NHANES 2011–2020.

Characteristic	OR (95% CI)	*P*-value	P for interaction
Age (years)			
<40	0.91 [0.58–1.42]	0.683	0.094
40–59	0.69 [0.49–0.97]	0.031	
≥60	0.57 [0.42–0.79]	0.004	
Ethnicity			
Hispanic	0.87 [0.58–1.30]	0.494	0.137
Non-Hispanic White	0.55 [0.40–0.76]	<0.001	
Non-Hispanic Black	0.7 [0.47–1.01]	0.052	
Other	0.72 [0.46–1.12]	0.143	
Alcohol drinking			
Non-drinking	0.58 (0.45–0.76)	<0.001	0.118
Low to moderate drinking	0.67 (0.31–1.47)	0.32	
Heavy drinking	0.81 (0.53–1.22)	0.31	
Smoking status			
Smoker	0.78 [0.52–1.16]	0.216	0.089
Non-smoker	0.57 [0.43–0.75]	<0.001	
Body mass index (kg/m2)			
<24.9	0.66 [0.45–0.97]	0.036	0.071
25–29.9	0.59 [0.42–0.83]	0.002	
≥30	0.52 [0.37–0.73]	<0.001	

Each stratification was adjusted for age + ethnicity + marital status + poverty income ratio + hypertension + BMI + smoking status+ alcohol drinking. The strata variable was not included when stratifying by itself. PIR, poverty income ratio. DI-GM, dietary index for gut microbiota; NHANES, National Health and Nutrition Examination Survey; OR, Odds Ratio; CI, Confidence Interval.

## Discussion

In this cross-sectional analysis of NHANES 2011–2020 data, which includes a representative sample of U. S. adults, we evaluated the relationship between DI-GM and breast cancer prevalence among women. We found that higher DI-GM scores were associated with a lower risk of breast cancer, even after adjusting for major confounders (e.g., age, body mass index, smoking status, alcohol drinking, and other demographic factors). The magnitude of the association remained consistent when DI-GM was modeled as a continuous predictor or by quantiles. Subgroup analyses showed that the inverse association between DI-GM and breast cancer was particularly notable among individuals aged ≥60 years, Non-Hispanic White people, non-smokers, and those with higher BMI, although no statistically significant interactions were observed. These findings suggest that a diet promoting gut microbiota health may play a protective role against breast cancer across diverse demographic and health profiles.

To our knowledge, this is one of the first studies to examine a gut-microbiota–oriented dietary index in relation to breast cancer. Previous epidemiologic studies have mostly examined broader diet patterns. A meta-analysis of observational studies found a significant inverse association between whole grain intake and breast cancer risk in the case–control studies, but not in the cohort studies (19). Cohort studies are generally considered stronger because they are prospective, collecting exposure data before the disease develops, which minimizes recall bias (20). In a prospective cohort study, refined grain food intake in the early adulthood was inversely associated with postmenopausal breast cancer, but not overall or premenopausal breast cancer risk (21). Another meta-analysis showed an inverse association between fiber intake and breast cancer risk, even following subgroup analysis based on study design (22). He et al. reported that intake of fermented dairy products led to a reduced risk of breast cancer in postmenopausal women, but not in premenopausal population (23). Romanos-Nanclares et al. (24) revealed that adherence to an overall plant-based diet index was associated with lower risk of breast cancer. Adherence to a Mediterranean dietary pattern (rich in fiber, polyphenols and anti-inflammatory nutrients) has been associated with beneficial shifts in gut microbiota and reduced breast cancer incidence (25). Meta-analysis of observational studies indicated that there is a positive association between processed/ unprocessed meat/high fat diet consumption and breast cancer risk (26, 27). In one study, obese women on chemotherapy showed enrichment of taxa such as *Collinsella*, *Roseburia* and *Prevotella* compared to non-obese cases. Therefore, obesity may alter the gut microbiota and influence symptom burden in women with breast cancer (28). Collectively, these findings emphasize the growing evidence linking diet, particularly components that influence gut microbiota composition, with breast cancer risk. While prior studies have focused on individual dietary elements or broader patterns, our study uniquely highlights the relevance of a gut-microbiota–oriented dietary index. The consistent inverse associations observed with fiber, whole grains, fermented dairy, and plant-based diets support the biological plausibility of our findings.

Several biological mechanisms may underlie the inverse DI-GM–breast cancer association. A high-DI-GM diet favors microbes that ferment fiber into short-chain fatty acids (SCFAs). SCFAs such as butyrate and propionate have anti-inflammatory and anti-proliferative effects (maintaining intestinal barrier integrity, modulating immunity, and inhibiting tumor cell growth). By contrast, a dysbiotic microbiota may produce procarcinogenic metabolites (e.g., secondary bile acids, reactive oxygen species) that induce DNA damage and chronic inflammation (29). Emerging evidence supports that gut microbial dysbiosis can drive breast carcinogenesis by modulating systemic immunity and inflammation. Zhang et al. (30) used Mendelian randomization to show that specific gut microbial metabolic pathways are causally linked to breast cancer risk, with part of this effect mediated by immune cell subsets (CD4^+CD8^+ leukocytes) (30). Consistent with an immune mechanism, Wang et al. (31) demonstrated in mice that remodeling the gut microbiota (via the herbal compound Huaier) enhanced breast tumor immunity. Huaier supplementation increased *Akkermansia* abundance and its metabolite butyrate, which synergized with a CDK4/6 inhibitor to boost CD8^+ T-cell infiltration and tumor suppression (31). These studies illustrate that dysbiosis (e.g., loss of beneficial taxa or gain of pro-tumor microbes) can alter breast cancer immune surveillance through cytokine signaling and T-cell dynamics. Notably, Lasagna et al. (32) found that estrogen-receptor+ patients with aromatase inhibitor resistance had higher gut microbial diversity and enrichment of *Veillonella*, accompanied by elevated IL-17 in tumors with low lymphocyte infiltrates. Dysbiosis can also increase gut permeability, allowing microbial endotoxins (e.g., lipopolysaccharide) to enter circulation and elicit chronic inflammation, a known cancer-promoting state (33). Gut microbes also intersect with hormonal pathways in breast cancer. Hillege et al. (34) reported that tamoxifen therapy in postmenopausal ER^+ patients modestly increased gut microbial richness, although overall community structure remained stable. This suggests that estrogen blockade may subtly shift gut ecology without wholesale dysbiosis. These data imply that endocrine therapies influence the gut estrobolome and associated immune milieu. For example, microbiota capable of β-glucuronidase and sulfatase activity can reactivate estrogens, potentially fueling hormone-dependent tumor growth. Simultaneously, microbial products like IL-17–inducing signals may promote chronic inflammation. Thus, hormone–microbiota crosstalk emerges as a dual pathway: altered gut estrogen metabolism (the estrobolome) and microbiota-driven inflammation both may modulate breast tumor proliferation and therapy response (32, 34). Moreover, microbial metabolites like butyrate can modulate host gene expression via epigenetic mechanisms. Butyrate is a histone deacetylase inhibitor that may upregulate tumor suppressor genes and promote apoptosis in mammary tissue (35). Together, these pathways suggest that diets conducive to a balanced microbiota could enhance tumor-suppressive processes and inhibit tumor-promoting signals.

Emerging data from both human and animal studies support a diet–microbiota–metabolite axis linking gut fermentation to breast cancer biology. For example, in HER2/neu transgenic mice a diet supplemented with broccoli sprouts (sulforaphane) and green tea polyphenols markedly altered the gut flora (increasing SCFA-producing genera like *Lachnospiraceae* and *Adlercreutzia*) and raised plasma short-chain fatty acids (notably propionate and isobutyrate) (36). In humans, dietary fiber interventions significantly reduced serum estrone and estradiol levels (37). It is worth noting that clinical data can be complex. For instance, one small cohort of postmenopausal patients found no simple correlation between self-reported fiber intake and circulating estradiol/estrone (38). These inconsistencies likely reflect multiple confounders in free-living humans.

Higher DI-GM scores were significantly associated with elevated urinary enterolignan metabolites (enterodiol and enterolactone), which are considered biomarkers of greater gut microbial richness/diversity. In other words, each 1-point higher DI-GM predicted small but significant increases in enterodiol/enterolactone, consistent with more lignan-metabolizing bacteria (11). As yet, however, no published study has directly tested DI-GM against stool microbiome sequencing or measured fecal SCFAs. All evidence of “validation” comes from biomarkers or epidemiologic inference. In practice, DI-GM has been applied mainly in large US and European cohorts: higher scores consistently predict lower odds of metabolic and chronic diseases metabolic syndrome (39), type 2 diabetes (13), cardiovascular disease (40), gastrointestinal cancers (41), and even women infertility (42). These associations lend construct validity, but they do not substitute for microbiome profiling. Limitations of DI-GM include its binary scoring (above/below median intake per food) and the fact that it was derived and tested chiefly in US (NHANES) or Western populations. For example, green tea intake was effectively zero in NHANES, so the score ranged only 0–13 (11).

Despite the robust findings, several limitations must be acknowledged. First, it is important to note that this was a cross-sectional study using self-reported history of breast cancer. As such, the results describe associations with prevalent breast cancer rather than incident cases, and causality cannot be inferred. Additionally, the potential for reverse causation exists individuals who have previously been diagnosed with breast cancer may have modified their dietary intake (e.g., adopting a healthier or more gut-friendly diet), which could bias the observed association between DI-GM and cancer history. Longitudinal data and prospective designs are needed to clarify the temporal sequence and minimize this concern. Second, dietary intake was self-reported through 24-h recalls, which are subject to recall bias and measurement errors. Third, although we adjusted for multiple important confounders, residual confounding due to unmeasured variables, such as genetic susceptibility or antibiotic use, is possible. Important breast cancer risk factors such as genetic predisposition (e.g., BRCA mutations or detailed family history) were not available, which may confound the observed associations. Additionally, while NHANES collects data on dietary supplement use, the frequency, duration, and specific types of probiotics were not consistently captured, limiting our ability to evaluate the potential modifying effects of probiotic or supplement intake. Fourth, DI-GM serves as an indirect proxy for gut microbiota health based on dietary intake and does not provide direct measures of microbiota composition or function, limiting mechanistic interpretation. Fifth, breast cancer history was self-reported and not confirmed by medical records, introducing the potential for misclassification. Sixth, the number of individuals with breast cancer in our sample was relatively small, which may reduce the statistical power and limit the generalizability of our findings to broader populations. Strengths of this study include the large, nationally representative sample, the comprehensive adjustment for key covariates, the consistent findings across multiple subgroups, and the novel use of a validated dietary index tailored to gut microbiota health.

Future research should aim to build on these findings through several directions. Prospective longitudinal studies are necessary to establish the temporal and potentially causal relationships between gut microbiota-targeted dietary patterns and breast cancer risk. Integrating dietary data with direct measures of gut microbiota (such as 16S rRNA gene sequencing, metagenomics, or metabolomics) could provide richer insights into how specific bacterial taxa and functional pathways mediate breast cancer risk. Randomized controlled trials evaluating the effects of microbiota-modulating interventions, including probiotics, prebiotics, synbiotics, and specific dietary patterns, on breast cancer prevention and progression would be particularly valuable. Moreover, mechanistic studies using *in vitro* and *in vivo* models could help elucidate the specific microbial metabolites and immune pathways involved. Lastly, future investigations should consider stratification by breast cancer subtype (e.g., estrogen receptor-positive, HER2-positive, triple-negative) to determine whether microbiota-related dietary interventions may offer subtype-specific preventive benefits.

## Conclusion

In this nationally representative sample of U.S. adults, higher adherence to a DI-GM was significantly associated with lower odds of having a self-reported history of breast cancer even after adjusting for key confounders such as age, ethnicity, hypertension, BMI, and smoking status. Stratified analyses further confirmed the robustness of this inverse relationship across different age, ethnicity, smoking, and BMI categories, although no significant interactions were detected.

## Data Availability

The datasets presented in this study can be found in online repositories. The names of the repository/repositories and accession number(s) can be found below: https://wwwn.cdc.gov/nchs/nhanes.

## Footnotes

^1^ https://wwwn.cdc.gov/nchs/nhanes
